# JINXED: just in time crystallization for easy structure determination of biological macromolecules

**DOI:** 10.1107/S2052252523001653

**Published:** 2023-03-09

**Authors:** Alessandra Henkel, Marina Galchenkova, Julia Maracke, Oleksandr Yefanov, Bjarne Klopprogge, Johanna Hakanpää, Jeroen R. Mesters, Henry N. Chapman, Dominik Oberthuer

**Affiliations:** a Center for Free-Electron Laser Science CFEL, Deutsches Elektronen-Synchrotron DESY, Notkestr. 85, 22607 Hamburg, Germany; b Deutsches Elektronen-Synchrotron DESY, Notkestr. 85, 22607 Hamburg, Germany; c Institut für Biochemie, Universität zu Lübeck, Ratzeburger Allee 160, 23562 Lübeck, Germany; d Department of Physics, Universität Hamburg, Luruper Chaussee 149, 22761 Hamburg, Germany; e The Hamburg Center for Ultrafast Imaging, Universität Hamburg, Luruper Chaussee 149, 22761 Hamburg, Germany; Uppsala University, Sweden

**Keywords:** TapeDrive 2.0, JINXED, protein crystallization, protein dynamics

## Abstract

Macromolecular crystallography is a well established method in the field of structural biology and has led to the majority of known protein structures to date. After focusing on static structures, the method is now under development towards the investigation of protein dynamics through time-resolved methods. These experiments often require multiple handling steps of the sensitive protein crystals, *e.g.* for ligand-soaking and cryo-protection. These handling steps can cause significant crystal damage, and hence reduce data quality. Furthermore, in time-resolved experiments based on serial crystallography, which use micrometre-sized crystals for short diffusion times of ligands, certain crystal morphologies with small solvent channels can prevent sufficient ligand diffusion. Described here is a method that combines protein crystallization and data collection in a novel one-step process. Corresponding experiments were successfully performed as a proof-of-principle using hen egg-white lysozyme and crystallization times of only a few seconds. This method, called JINXED (Just IN time Crystallization for Easy structure Determination), promises high-quality data due to the avoidance of crystal handling and has the potential to enable time-resolved experiments with crystals containing small solvent channels by adding potential ligands to the crystallization buffer, simulating traditional co-crystallization approaches.

## Abstract 2

In this work, a method is developed and tested to obtain protein crystals on-the-fly, before immediately probing them with X-rays. The resulting crystals proved to be useful for serial crystallography using the CFEL TapeDrive and yielded high-quality datasets with useful resolution to 1.71 Å.

## 1. Introduction

Protein crystallization was first described more than 150 years ago (Giegé, 2013 ▸; McPherson, 1991 ▸; McPherson & Gavira, 2014 ▸) and has enabled the structure determination of bio­logical macromolecules at an atomic level (Dickerson, 2005 ▸). Notwithstanding the recent success of single-molecule cryoEM, crystallography is still the method of choice in many areas of structural biology and structure-based drug discovery. The crystallization of biological macromolecules is a tedious process owing to the complex structure, dynamic behaviour and complex surface charge distribution of said molecules. Even after many decades of research it is not completely understood. Once crystals of good quality are obtained, the standard single-crystal rotational data collection at cryogenic temperatures requires a lot of crystal handling from fishing, ligand soaking, cryo-protecting to flash-cooling. These various handling steps often damage the sensitive protein crystal, reducing its quality and diffraction capabilities, and hence limit the chances of collecting high-resolution diffraction data (Dobrianov *et al.*, 1999 ▸). Thus, a lot of research has been focused on mitigating these effects, mainly through the development of *in situ* crystallization approaches, aided in part by the coming of age of microfluidic methods from the middle of the 1990s onwards (Hansen & Quake, 2003 ▸; Heymann *et al.*, 2014 ▸; Perry *et al.*, 2013 ▸; De Wijn *et al.*, 2019 ▸; Yadav *et al.*, 2005 ▸). With serial crystallography (Barends *et al.*, 2022 ▸; Boutet *et al.*, 2012 ▸; Chapman *et al.*, 2011 ▸) and subsequent room-temperature data collection coming into play, not only has the required crystal size decreased to micrometre and sub-micrometre dimensions, but also the required handling steps have been drastically reduced since fishing, cryo-protection and flash cooling have become unnecessary. Furthermore, structural biology no longer exclusively focuses on static structures (Martin-Garcia, 2021 ▸) but – as biology naturally implies dynamical interactions between molecules – aims to reveal protein dynamics, especially when it comes to protein–ligand interactions. Serial crystallography offers the possibility to study these interactions at an atomic level in time-resolved experiments (Brändén & Neutze, 2021 ▸; Mehrabi *et al.*, 2019 ▸; Pande *et al.*, 2016 ▸; Stagno *et al.*, 2017 ▸; Tenboer *et al.*, 2014 ▸), *e.g.* by mixing the crystalline slurry with substrate-containing solution before probing the mixture with X-rays after a defined time delay (Beyerlein *et al.*, 2017 ▸). However, chemical mixing is limited by crystal and solvent-channel sizes, accessibility of the binding site, type of binding principle (lock-and-key versus induced fit), ligand solubility, ligand size and speed of diffusion (solute viscosity), all of which are not straightforward. A further set of challenges originates from sample delivery: crystals tend not to be perfectly suspended in the microcrystalline slurry, especially at lower but also higher solution viscosities and also when the crystals are larger than about a micrometre (Berntsen *et al.*, 2019 ▸). There have been solutions to this crystal-settling problem (Lomb *et al.*, 2012 ▸), but those require longer fluidic lines, introducing potential sources of error, since the crystals can settle in capillaries as well as stick to their walls and cause clogging of the lines (Clabbers *et al.*, 2021 ▸). Automatic sample exchange for crystalline slurries is also not straight forward, whereas for non-crystalline samples established automated sample dispensing systems (‘auto-sampler’), such as those used in HPLC systems and at SAXS beamlines, could be used. It would thus be of great benefit to grow the crystals only when needed, right before the sample is introduced into the X-ray focus: just in time. This would solve many challenges such as crystal damage, crystal soaking as well as sample delivery, and open completely new possibilities in time-resolved structural biology and structure- or fragment-based drug discovery. To that end, we developed a method for crystallization on-the-fly using the CFEL TapeDrive (Beyerlein *et al.*, 2017 ▸; Zielinski *et al.*, 2022 ▸) which yields crystals just in time for easy structure determination of biological macromolecules or JINXED (Just IN time Crystallization for Easy structure Determination). We present here the first structures obtained using JINXED at four different crystallization time points.

## 2. Methods

### 2.1. Protein sample and crystallizing solution

Hen egg-white lysozyme (Sigma–Aldrich) was prepared to a concentration of 126 mg ml^−1^ in 50 m*M* acetate buffer pH 3.5. The crystallizing agent contained 0.1 *M* sodium acetate, pH 4.6, 2.7 *M* NaCl, 15%(*w*/*v*) PEG4000, 6%(*v*/*v*) ethyl­ene glycol.

### 2.2. TapeDrive nozzle

**Figure 1:**
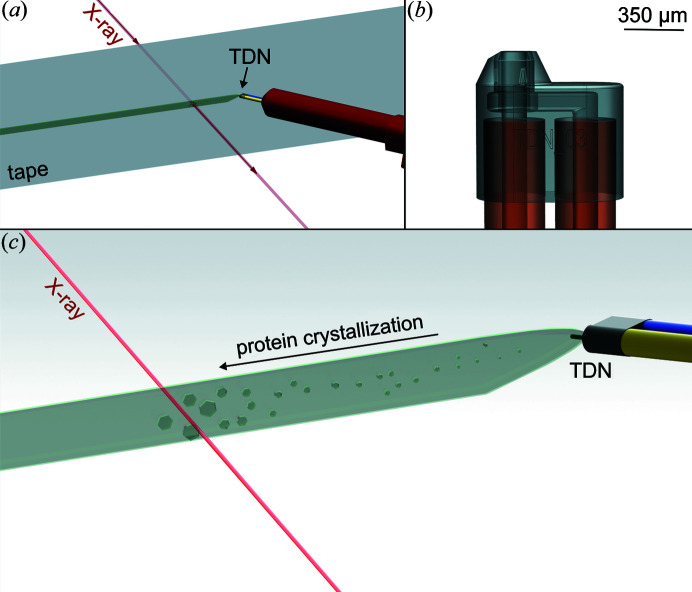
Drawings of (*a*) the sample environment overview with the TapeDrive nozzle (TDN), tape, sample line and X-ray beam; (*b*) TDN with inner (left) and outer mixing (right) channels; (*c*) JINXED method with TDN incorporating the protein solution channel (yellow) and crystallizing agent channel (blue). Due to mixing within the sample line on the tape, protein crystallization can be observed.

For the first TapeDrive prototype (Beyerlein *et al.*, 2017 ▸; Zielinski *et al.*, 2022 ▸), the sample was deposited onto the tape using polished fused silica fibres with inner diameters ranging from 50 to 180 µm and outer diameters of 360 µm. For mixing on the tape, nozzle-in-nozzle assemblies, derived from developments for double flow-focusing nozzles (Oberthuer *et al.*, 2017 ▸), were used. These assemblies were made by fitting a fused silica capillary with an outer diameter smaller than the inner diameter of the second capillary into the second capillary (Wang *et al.*, 2014 ▸). A three-way connector (IDEX) was used to decouple the outer and inner capillary. Using this connector, the relative position of the end of the inner capillary towards the opening of the outer capillary could be adjusted and thus reduce the contact time between sample and substrate before probing the mixture with X-rays for time-resolved experiments (Beyerlein *et al.*, 2017 ▸). The manufacturing of this assembly was not only tedious, but also prone to error. Nano-precision 3D-printing of nozzles for liquid jet injection has been established in the past few years and is now widely used (Knoška *et al.*, 2020 ▸; Nelson *et al.*, 2016 ▸; Wiedorn *et al.*, 2018 ▸). For this study, where mixing of protein solution and crystallizing agent at defined time-steps was required, the design for a 3D-printed gas dynamic virtual nozzle (GDVN; Knoška *et al.*, 2020 ▸) was modified and optimized for deposition of sample onto a running tape and simultaneous mixing with a second solution (in this case the crystallizing agent). Since gas-focusing is not needed for sample deposition on the tape, the gas channel was replaced by the mixing channel and mixing on the tape was conducted in the same manner as the fast-mixing setting of the original TapeDrive (Beyerlein *et al.*, 2017 ▸). The adapted and modified design was subsequently optimized for nano-precision 3D-printing (NanoScribe), resulting in the TapeDrive nozzle [TDN, Figs. 1 ▸(*b*) and S1 of the supporting information] as used in this study. However, the usage of the TDN is not limited to the JINXED method but can be used in both mixing and non-mixing modes for sample delivery with the CFEL TapeDrive. Depending on tape speed and TDN position relative to the X-ray focus, time delays between 50 ms and 100 s can be realized with this setup. The TDN is 970 × 450 × 860 µm in size (*l* × *w* × *h*, maximum dimensions from bottom to tip) and the orifice of the combined sample and mixing channel is 200 µm wide. The two fused silica capillaries feeding sample and crystallizing solution into the nozzle have inner diameters of 150 µm.

### 2.3. Data collection at P11 using JINXED

Sample delivery was performed by the CFEL TapeDrive 2.0 (manuscript in preparation), an updated version of the CFEL TapeDrive (Beyerlein *et al.*, 2017 ▸). The general description of sample delivery using a microfluidic controller as described by Zielinski *et al.* (2022 ▸) is still valid for TapeDrive 2.0 [see Figs. 1 ▸(*a*) and 2 ▸] which was optimized for fast installation at beamlines, ease of use, low sample consumption as well as more accurate tape movement. The tape (polypropylene, 15 µm thick, corona treated; Puetz-Folien) is oriented perpendicular to the X-ray beam and is drawn across the X-ray interaction region. For the JINXED method this was just slightly modified: the protein solution was placed in a reservoir and transported to the TDN using an Elveflow OB1 flow controller. The reservoir was connected directly to this controller and pressurized by the controller and not indirectly through a plunger. A microfluidic flow-rate sensor (Elveflow, France) was placed in line after the reservoir to provide feedback on the flow rate corresponding to a certain input pressure. The nozzle, as described above, was connected to the microfluidic tubing using standard HPLC connectors (IDEX, USA). A second reservoir, containing the crystallizing agent, was connected to the nozzle in the same way, using a different channel at the OB1 flow controller for independent control of both flow rates. The reservoirs, standard micro-reaction tubes with either 1.5 or 15 ml volumes, can be placed in a heating/cooling block or in a shaker for such tubes (*e.g.* Eppendorf ThermoMix C) for prolonged sample integrity. Use of fused silica fibres (Polymicro, USA) with an inner diameter of 150 µm as a connection between the 3D-printed nozzle and the sample reservoirs ensured a smooth sample flow with as little restriction and clogging as possible. The protein and crystallizing solution were mixed in a 1:1 ratio at a flow rate of 1 µl min^−1^, resulting in protein crystallization [Figs. 1 ▸(*c*) and S2] . In Video S1 of the supporting information, a 2 µl droplet of lysozyme solution (126 mg ml^−1^ in 50 m*M* acetate buffer pH 3.5) and a 2 µl droplet of the crystallizing agent [0.1 *M* sodium acetate, pH 4.6, 2.7 *M* NaCl, 15%(*w*/*v*) PEG4000, 6%(*v*/*v*) ethyl­ene glycol] were pipetted on a glass slide next to each other and subsequently mixed using a pipette tip, which resulted in instantaneous crystallization. The large amount of nuclei/crystals both in the microscope setup as well as during the experiment at the beamline prevented precise size measurements of the crystals. Using the mixture described, the crystals grew to a maximum size of 38 × 38 µm within 4 min. Therefore crystals from earlier time points must be smaller. The nozzle-to-beam distance was varied from 2 to 8 mm (at a tape speed of 1 mm s^−1^) to probe crystallization times of 2, 4, 6 and 8 s. The humidity in the sample environment was not controlled, the relative humidity in the experimental hutch of the beamline fluctuated between 20 and 54% during the experiment. X-ray data collection was carried out at beamline P11 (PETRA III, DESY, Hamburg) using 12.0 keV photon energy X-rays focused to a spot size of 4 × 9 µm (*w* × *h*) with a flux of 8.6 × 10^12^ photons s^−1^. *Raddose-3D* (Zeldin *et al.*, 2013 ▸) was used to estimate the dose, assuming the crystal size range 1 × 1 × 1 µm to 5 × 5 × 5 µm. The script given in the supporting information resulted in a dose range from 0.23 to 0.30 MGy.

Data were continuously collected for each dataset using an EIGER2 X 16M detector at 130 Hz frame rate, providing an exposure time of 7.69 ms. Feedback about hit und indexing rates of the incoming data was provided by the *OnDA* software package (Mariani *et al.*, 2016 ▸).

### 2.4. Data processing

Raw data files were processed with *CrystFEL* (version 0.9.1; White *et al.*, 2012 ▸, 2016 ▸) using custom scripts. In *indexamajig*, the option --peaks = peakfinder8 was used to identify individual ‘hits’ from the complete set of collected diffraction patterns. The complete set was defined by an automatically generated list of files. Detected ‘hits’ were then indexed using *XGANDALF* (Gevorkov *et al.*, 2019 ▸) and integrated. The geometry input file was adapted for the photon energy and detector distance from previous experiments at P11. The resulting stream-files were merged into the point group 4/*mmm* using *partialator* and the figures of merit were calculated using *compare_hkl* and *check_hkl*, all part of the *CrystFEL* package. The .mtz files for crystallographic data processing were generated from *CrystFEL* merged reflection data files using *F2MTZ* within the *CCP4* suite (Winn *et al.*, 2011 ▸). Fig. S3(*b*) was generated with custom python script, inspired by figure 8 in the work by Martin-Garcia *et al.* (2017 ▸).

The starting model for refinement was a lysozyme structure (PDB entry 6ftr; Wiedorn *et al.*, 2018 ▸) for all datasets and *Phenix* (Liebschner *et al.*, 2019 ▸) was used for refinement and model validation. *R*_free_ flags were generated using *phenix.reflection_file_editor* and the same set of *R*_free_ flags was used for all datasets in this study. In *phenix.refine* (Afonine *et al.*, 2012 ▸) *B* factors of the starting model were set to 20.0 for the first round of refinement, followed by visual inspection of the model and maps using *Coot* (Emsley *et al.*, 2010 ▸). Further iterative cycles of refinement included TLS, optimization of X-ray/stereochemistry and X-ray/ADP weights as well as manual model building in *Coot. MolProbity* (Williams *et al.*, 2018 ▸) was used for validation of the final model. Figures were generated using *PyMOL*, which was also used for the alignment of structures and r.m.s.d. calculations.

## 3. Results

Diffraction from protein crystals was observed first at 2 s after mixing the protein solution and crystallizing agent, manifesting the proof-of-principle of simultaneous protein crystallization and diffraction data collection, named JINXED. After 2 s of mixing time (at a nozzle-to-X-ray focus distance of 2 mm and a tape speed of 1 mm s^−1^), 40.6% of all recorded detector frames contained a ‘hit’ and 21.1% of those hits contained indexable patterns. After 4 s of mixing time (at a nozzle-to-X-ray focus distance of 4 mm and a tape speed of 1 mm s^−1^) the hit rate increased to 43.6%, whereas the indexing rate decreased to 18.6%. 6 s of mixing time (at a nozzle-to-X-ray focus distance of 6 mm and a tape speed of 1 mm s^−1^) resulted in a hit rate of 83.1% and an indexing rate of 42.1%. Extending the crystallization time to 8 s (at a nozzle-to-X-ray focus distance of 8 mm and a tape speed of 1 mm s^−1^) led to an increase of the hit rate to 94.8% and a decrease of the indexing rate to 25.9%. Detailed data collection and refinement statistics can be found in Table 1 ▸. A representative diffraction pattern (from the 8 s dataset, exemplary for all datasets) is shown in Fig. S3(*a*), as well as a plot of the mean radial intensity from the 2 s dataset, (only hits) against *Q* (1/*d*, in nm^−1^), including the error in the mean radial intensity (the standard deviation of the mean radial intensity) in Fig. S3(*b*).

For the dataset recorded at 4 s crystallization time, a total of 32 418 indexed lattices could be found. For better comparability of the four datasets, all were cut to 32 418 randomly selected indexed lattices prior to calculating the figures of merit (Table 1 ▸). The resolution cut-off was chosen individually for each dataset corresponding to CC* > 0.5. Between the four datasets, the resolution differs only by 0.07 Å. The overall signal-to-noise ratio (SNR) increases slightly with increasing crystallization time from 9.57 (2 s) to 10.76 (4 s), 10.21 (6 s) and 11.93 (8 s). A similar trend can be seen for the Wilson *B* factor, which is known to indicate the degree of order within the crystal. The continuous increase from 19.69 (2 s) to 23.47 (8 s) has not been correlated with increasing size in the existing literature yet and requires further studies to validate a true correlation.

**Figure 3:**
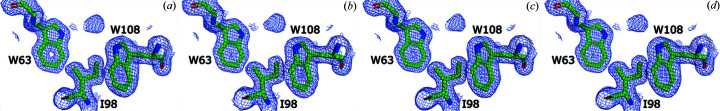
Electron-density maps (contour level σ = 1.0) with models of residues W63, I98, W108 for crystallization times of (*a*) 2 s, (*b*) 4 s, (*c*) 6 s and (*d*) 8 s.

**Figure 4:**
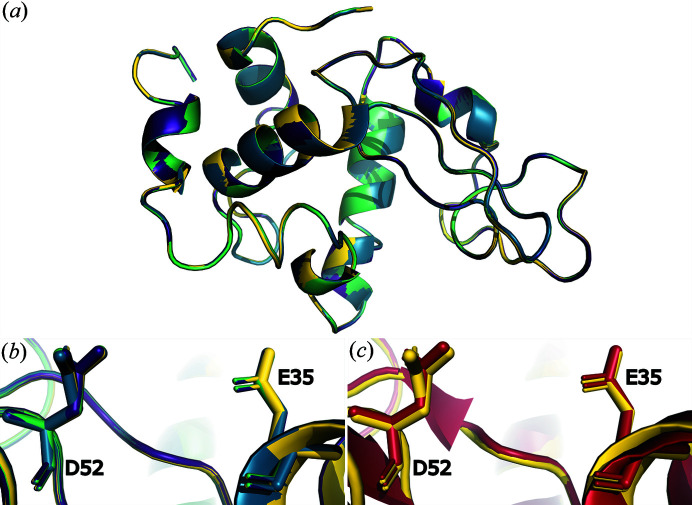
Structure overlay of (*a*) structures from all datasets with 2 s (violet), 4 s (blue), 6 s (green) and 8 s (yellow) crystallization times; (*b*) residues D52 and E35 from all structures from all datasets with 2 s (violet), 4 s (blue), 6 s (green) and 8 s (yellow) crystallization times; (*c*) residues D52 and E35 from the dataset with 8 s crystallization time (yellow) and the PDB entry6ftr model (red).

As shown in Fig. 3 ▸, all datasets yielded 2*F*_o_ − *F*_c_ electron density maps (contour level σ = 1.0) of very similar quality, and no clear trend between map quality and crystallization time could be observed. Additionally, *F*_o_ − *F*_o_ maps were calculated for each crystallization time combination (2–4 s, 2–6 s, 2–8 s, 4–6 s, 4–8 s, 6–8 s) but showed no difference at σ ≥ 5.0 and only minor differences at σ ≥ 4.0, which we identified as noise since none were related to the model. Difference maps at σ = 3.0 are shown in Fig. S4. Refinement (from the starting model: PDB entry 6ftr; Wiedorn *et al.*, 2018 ▸) resulted in *R*_work_ values of 0.171 (2 s), 0.164 (4 s), 0.166 (6 s) and 0.166 (8 s) with no clear tendency with increasing crystallization time. *R*_free_ values decreased slightly with increasing crystallization time from 0.205 (2 s), 0.202 (4 s), 0.195 (6 s) to 0.186 (8 s) which might be correlated with an increase in crystal size and corresponding increase in diffraction capability during the crystallization process. The refined structures of all four crystallization times were aligned with negligible RMSD (<0.05). Depicted in Fig. 4 ▸ is the overlay of all structure models [Fig. 4 ▸(*a*)], the overlay of the two residues, Glu35 and Asp52, forming the active site [Fig. 4 ▸(*b*)], and the active site overlay of the 8 s model and that from PDB entry 6ftr [Fig. 4 ▸(*c*)], showing no major differences between the structural models. The 8 s structure contains 68 water molecules, whereas the three structures of 2, 4 and 6 s crystallization times contain 72 water molecules. Among all four structures, only 59 water molecules appear to be identical whereas the 13 (for 2, 4 and 6 s) or 9 (for 8 s) remaining water molecules are located in random combinations for the different crystallization times. We can assume that the identical number of water molecules for three of the four models is only coincidental.

## 4. Conclusions

The experiments presented here using the JINXED method are the first of their kind and therefore provide a proof-of-principle for just in time crystallization and diffraction. The underlying idea was developed based on the crystallization behaviour of hen egg-white lysozyme (HEWL), which forms crystals immediately after mixing with the crystallizing agent, and the possibility for rapid mix-and-diffuse experiments with the TapeDrive setup (Beyerlein *et al.*, 2017 ▸). Diffraction from the HEWL microcrystals was detected just 2 s after mixing the protein and crystallizing agent, showing that HEWL forms crystals of sufficient size for SSX at a third-generation synchrotron within this time frame.

JINXED has the advantage that the sensitive microcrystals do not need to be handled in any form prior to data collection. This eliminates the occurrence of crystal damage caused by mechanical stress, which is often reported to limit the diffraction resolution of crystals (Dobrianov *et al.*, 1999 ▸).

**Figure 5:**
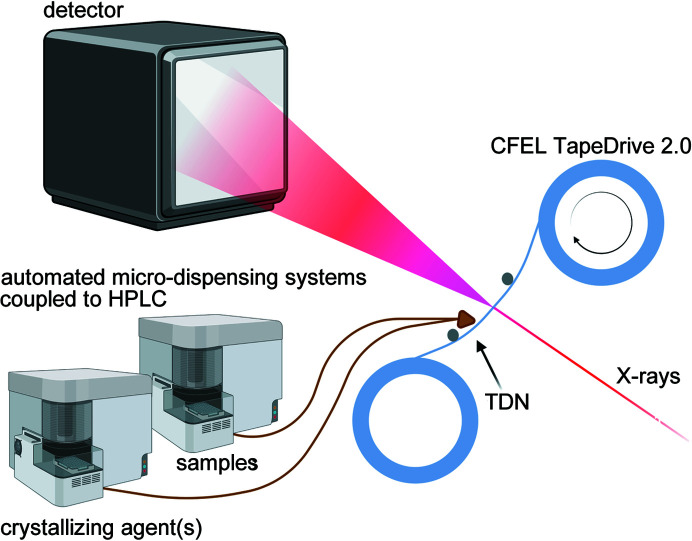
Schematic of a possible high-throughput setup showing two automated micro-dispensing systems for samples (*e.g.* protein mixed with compound) and crystallization agents, the sample delivery system CFEL TapeDrive 2.0 including the TDN, X-ray beam and detector.

Regarding the variety of crystallographic experiments, even time-resolved mix-and-diffuse experiments or inhibitor and fragment-binding studies can be conducted within our approach. This has the potential to solve the problem of insufficient diffusion saturation of the crystals for various reasons (see the Introduction[Sec sec1]), which are also faced by other data collection methods of serial time-resolved studies. Furthermore, it is less tedious than co-crystallization prior to the experiment. In principle, compounds for structure- or fragment-based drug design could be incubated with protein in, for example, 384 well SBS-format plates and an auto-sampler like those used in HPLC instruments, and then the protein-compound solutions could be dispensed one after the other to the TDN for JINXED and high-throughput data collection using TapeDrive 2.0 (Fig. 5 ▸). Similarly, for time-resolved enzyme studies, the substrate or analogues could be mixed with crystallizing agent. The resulting time delay would be equal to that of the crystallization time. However, at XFELs, crystals much smaller in size [few 100 nm in diameter (Gati *et al.*, 2017 ▸)] can be used and thus those of a sufficient quality could be available in a few milliseconds. In addition to mix-and-diffuse, it would also be compatible with optical reaction triggering and pH-jump experiments.

Nonetheless, JINXED experiments require fast crystallization of the protein sample. The screening for suitable crystallization conditions may be time-consuming and extensive, but this challenge is equally faced in protein crystallography in general (Chayen, 2004 ▸; Chayen & Saridakis, 2008 ▸). Once successful crystallization conditions are determined, optimization of the latter according to the general phase behaviour of proteins could lead to faster crystallization rates suitable for this approach. The focus of protein crystallization research has been, obviously, on the reliable production of large, well diffracting crystals, with few examples focusing on the directed generation of small crystals for solid-state NMR (Martin & Zilm, 2003 ▸), SFX and SSX (Beale *et al.*, 2019 ▸; Stohrer *et al.*, 2021 ▸; Tenboer *et al.*, 2014 ▸). JINXED could even be used to screen for crystallization conditions and crystallization times without the need for manual crystallization trials and visual assessment since this method can easily be automated using a high-throughput setup (Fig. 5 ▸) and would provide direct proof of crystalline properties. We can assume that many proteins could be crystallized rapidly if conditions were optimized towards the velocity of crystallization as reported by Santarsiero *et al.* (2002 ▸). However, a better understanding of the underlying physics of protein nucleation and crystallization, and further research in that direction is necessary, but way beyond the scope of this study. Again, the adaptation to X-ray free-electron-laser beamlines would lower the required crystal size, which may enable JINXED experiments for proteins that show slower growth rates. For SFX experiments it would be beneficial to use a sample delivery method that avoids the introduction of superfluous material to the beam, since this appears to be problematic for the high-intensity X-ray pulses produced by XFELs. For example, liquid jets generated by nozzles with integrated mixing devices (Knoška *et al.*, 2020 ▸) may be suitable for implementing JINXED at XFELs.

The variety of proteins suitable for JINXED experiments at SSX beamlines can be broadened by exploiting the possibility for prolonged mixing/crystallization times of up to several minutes using TapeDrive (Beyerlein *et al.*, 2017 ▸), which improves the general applicability of this method. Moreover, the addition of temperature control to the TapeDrive might facilitate rapid crystallization for proteins with temperature-dependent solubility. Note, multi-dimensional studies recently became popular in serial crystallography to investigate structural changes in response to temperature (Mehrabi *et al.*, 2021 ▸). These experiments could again benefit from the strategy presented in this study, since the crystals are directly grown in different environments and not confronted with environment changes after growth, reducing stress on the crystal and thus potentially increasing diffraction quality.

Due to the numerous advantages of JINXED as outlined above, this method should be further evaluated, including different proteins, setups and beamlines to assess the overall applicability of this novel approach. Even if the method is found to be suitable for a subset of proteins of biological or pharmaceutical relevance, it could be a game changer for high-output studies of enzyme dynamics and drug- and fragment-binding properties.

## Supplementary Material

Crystallization on a glass slide mediated by a pipette. DOI: 10.1107/S2052252523001653/zf5020sup1.mp4

Supporting figures and calculations. DOI: 10.1107/S2052252523001653/zf5020sup2.pdf

PDB reference: hen egg-white lysozyme 2s *in situ* crystallization, 8b3l

PDB reference: hen egg-white lysozyme 8s *in situ* crystallization, 8b3v

PDB reference: hen egg-white lysozyme 6s *in situ* crystallization, 8b3u

PDB reference: hen egg white lysozyme 4s *in situ* crystallization, 8b3t

## Figures and Tables

**Figure 2:**
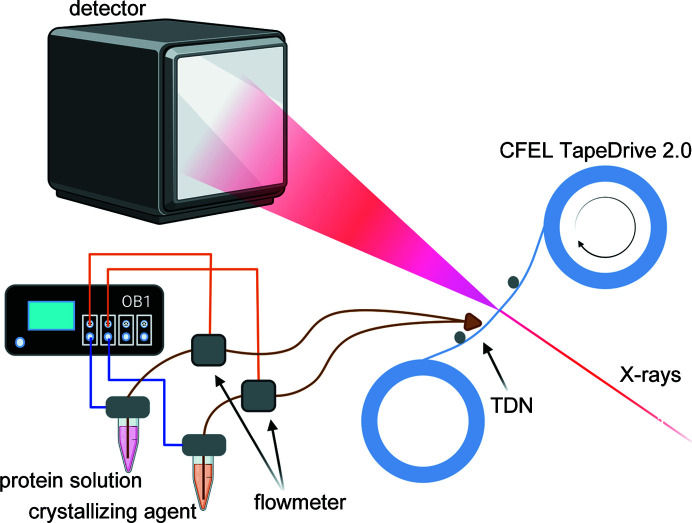
Schematic of the experimental setup showing the Elveflow controller OB1, microfluidic flow rate sensors (flowmeters), reservoirs with protein solution and crystallization buffer, the sample delivery system CFEL TapeDrive 2.0 including the TDN, X-ray beam and detector Eiger2 X 16M.

**Table 1:** Data-collection and refinement statistics for four different time points of the JINXED data collection

	Crystallization time			
	2 s	4 s	6 s	8 s
No. of collected images	600000	400000	400000	400000
No. of hits	243670	174523	332437	379300
Hit rate (%)	40.6	43.6	83.1	94.8
Indexed patterns	51 415	32 418	139 879	98 218
Indexing rate (%)	21.1	18.6	42.1	25.9
Dose (MGy)	0.23–0.30			
Space group	*P*4_3_2_1_2	*P*4_3_2_1_2	*P*4_3_2_1_2	*P*4_3_2_1_2
Unit cell parameters				
*a* = *b* (Å)	79.2	79.2	79.2	79.2
*c* (Å)	37.8	37.8	37.8	37.8
α = β = γ (°)	90.0	90.0	90.0	90.0

Statistics for 32418 indexed patterns				
Resolution (Å)	39.60–1.71 (1.74–1.71)	39.60–1.78 (1.81–1.78)	39.60–1.71 (1.74–1.71)	39.60–1.74 (1.77–1.74)
SNR	9.57 (0.38)	10.76 (0.3)	10.21 (0.37)	11.93 (0.41)
Completeness (%)	100.0 (100.0)	100.0 (100.0)	100.0 (100.0)	100.0 (100.0)
Total measurements	5641214	4947646	6834865	6080391
Unique reflections	13615 (674)	12126 (601)	13615 (674)	12939 (620)
Multiplicity	414.33 (36.8)	408.01 (54.7)	502.00 (25.6)	469.92 (57.8)
*R*_split_ (%)	13.15 (127.91)	10.22 (146.04)	11.67 (171.07)	9.26 (115.28)
CC_1/2_	0.980 (0.347)	0.992 (0.275)	0.989 (0.247)	0.992 (0.289)
CC*	0.995 (0.718)	0.998 (0.656)	0.997 (0.63)	0.998 (0.67)
Wilson *B* factor (Å^2^)	19.69	20.88	21.01	23.47

Refinement				
PDB entry	8b3l	8b3t	8b3u	8b3v
Resolution (Å)	39.6–1.71 (1.771–1.71)	39.6–1.78 (1.844–1.78)	39.6–1.71 (1.771–1.71)	39.6–1.74 (1.802–1.74)
No. of reflections	13258 (1092)	11776 (958)	13111 (996)	12748 (1167)
Reflections used for *R*_free_	1333 (119)	1181 (96)	1312 (97)	1277 (118)
*R*_work_	0.1706	0.1640	0.1658	0.1661
*R*_free_	0.2054	0.2022	0.1946	0.1860
No. of atoms				
Protein	1140	1140	1140	1140
Ligand/ion	50	50	50	50
Water	72	72	72	68
Ramachandran favoured (%)	98.43	97.64	99.21	99.21
Ramachandran allowed (%)	1.57	2.36	0.79	0.79
Ramachandran outliers (%)	0.00	0.00	0.00	0.00
R.m.s deviations				
Bond lengths (Å)	0.008	0.006	0.015	0.005
Bond angles (°)	0.83	0.80	1.19	0.71
Clashscore	5.64	3.47	4.34	4.34
Average *B* factor	25.04	28.99	28.47	29.66
Macromolecules	24.05	27.99	27.49	28.75
Ligands	49.68	56.0	56.40	54.76
Solvent	32.73	36.15	34.94	36.51
