# Supplementary material for: JINXED: just in time crystallization for easy structure determination of biological macromolecules
Source: IUCrJ. 2023 Mar 9;10(Pt 3):253–60. doi: 10.1107/S2052252523001653 (PMC10161778; doi:10.1107/S2052252523001653)
Supplement: Supplementary file 2 [file m-10-00253-sup2.pdf]

IUCrJ

Volume 10 (2023)

Supporting information for article:

JINXED: Just in time crystallization for easy structure determination of biological macromolecules

Alessandra Henkel, Marina Galchenkova, Julia Maracke, Oleksandr Yefanov, Johanna Hakanpää, Jeroen R Mesters, Henry N Chapman and Dominik Oberthuer

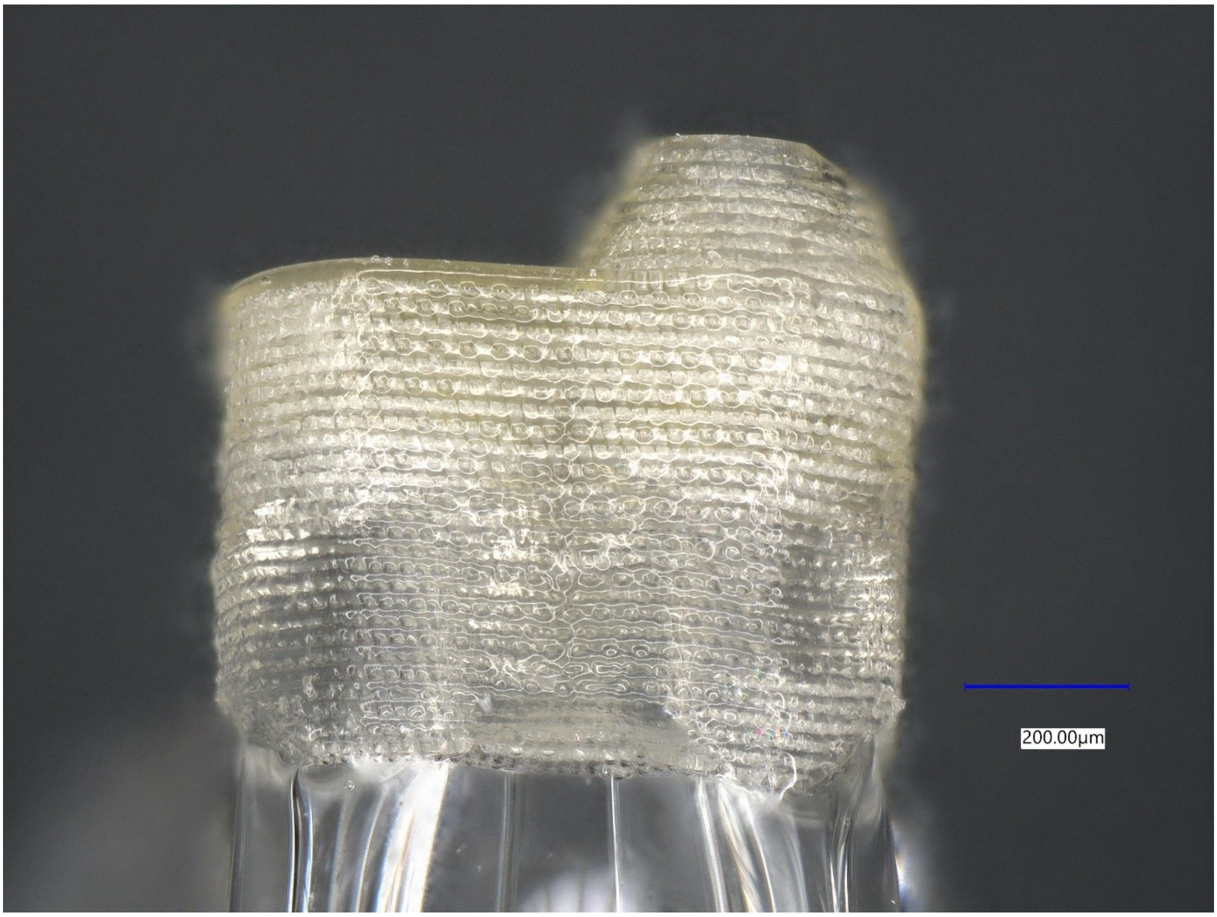

Figure S1: Microphotograph (depth image) of the TapeDrive nozzle (Keyence VHX-7000).

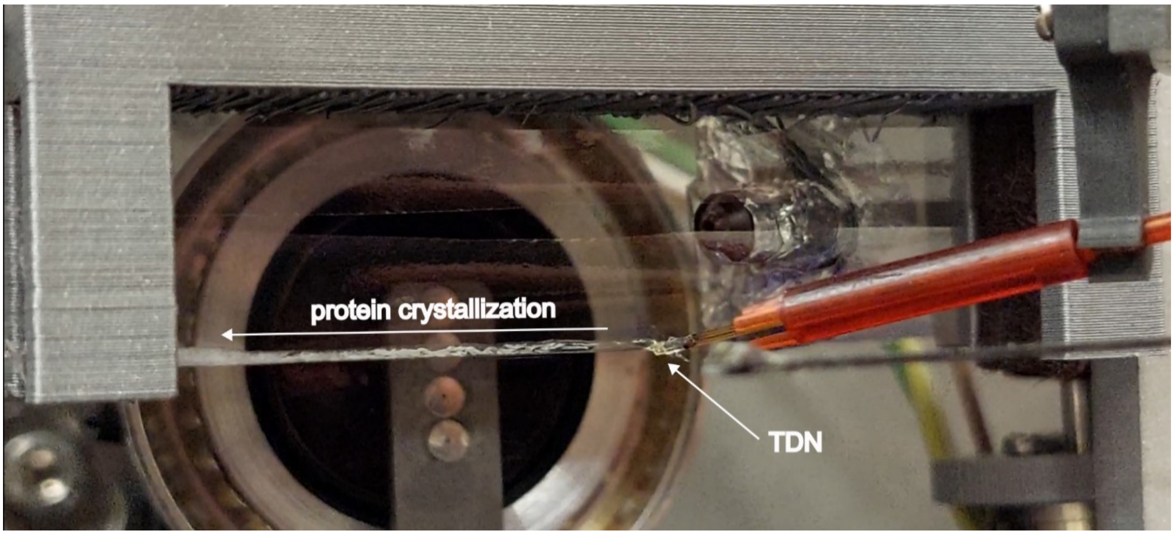

Figure S2: Photograph of JINXED at P11, DESY, HH. The 3D-printed TapeDrive nozzle deposits the protein solution and crystallizing agent onto the tape where mixing occurs subsequently. The white clouding within the sample line indicates protein crystallization.

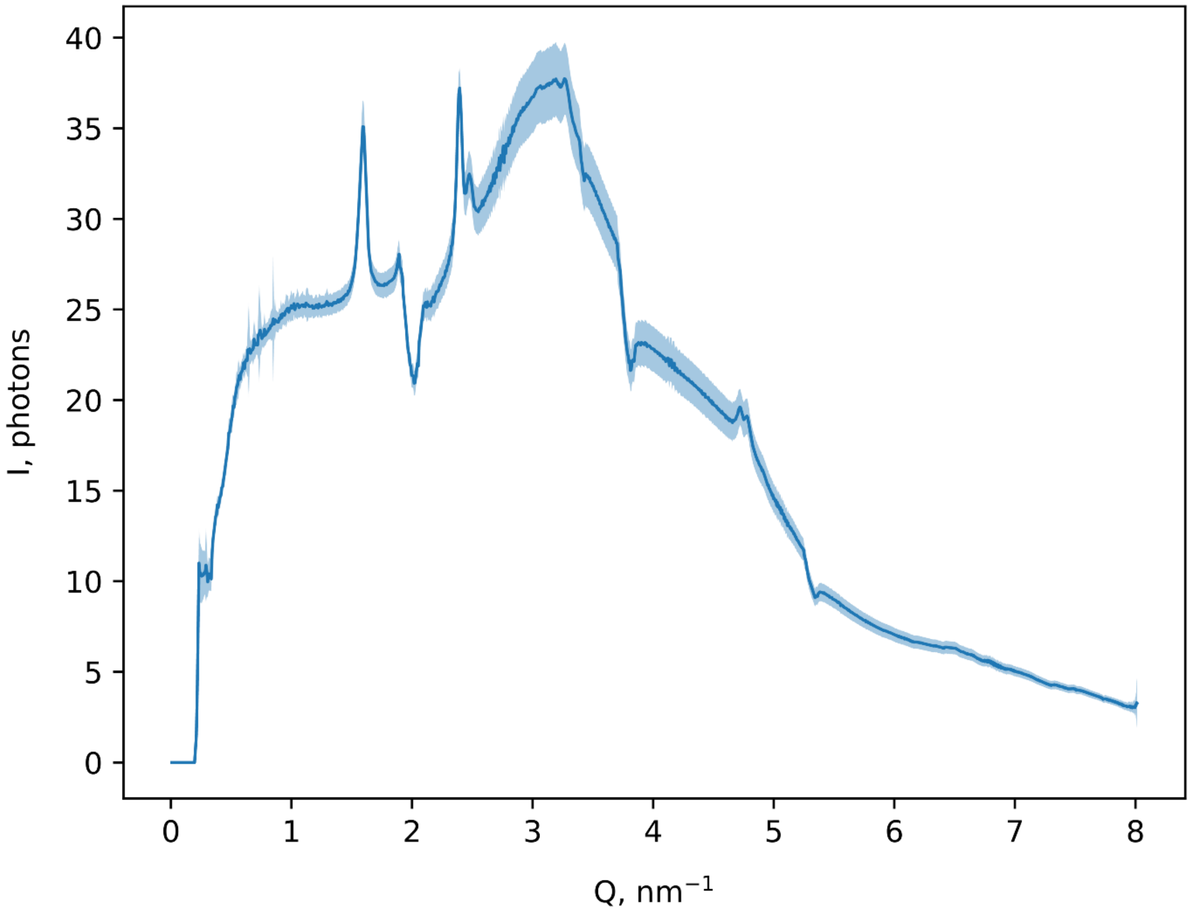

Figure S3: Plot of the mean radial intensity from the 2s dataset (only hits) against Q (1/d) in nm-1. The error in mean radial intensity (the standard deviation of the mean radial intensity) is shown as semi-transparent blue region behind the blue line representing the mean radial intensity.

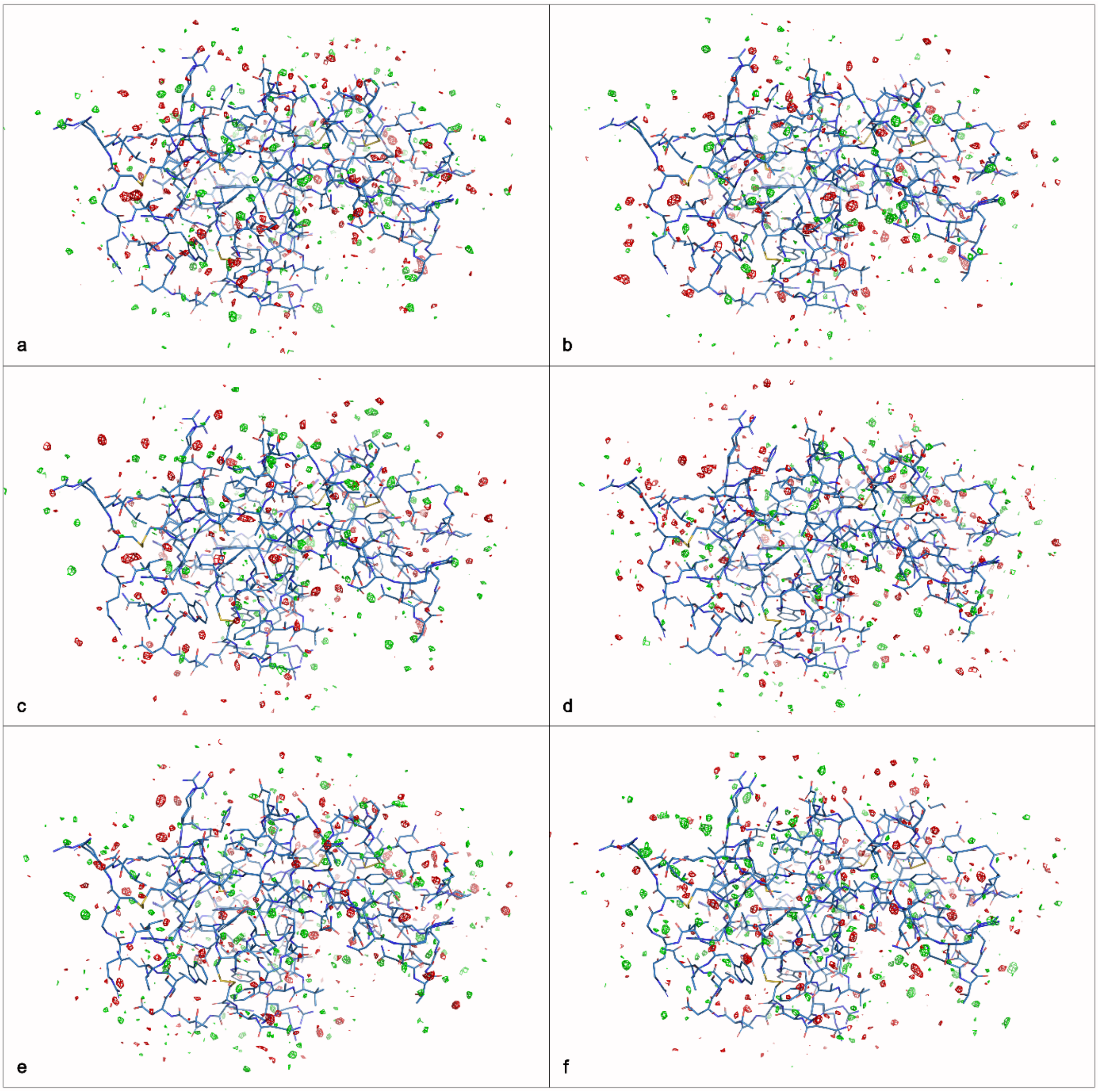

Figure S4: Difference maps (σ = 3.0) of the different time points of JINXED: a) Fo(2s)-Fo(4s) with 2s-model, b) Fo(2s)-Fo(6s) with 2s-model, c) Fo(2s)-Fo(8s) with 2s-model, d) Fo(4s)-Fo(6s) with 4s-model, e) Fo(4s)-Fo(8s) with 4s-model, f) Fo(6s)-Fo(8s) with 6s-model. Positive density is depicted in green, negative density is depicted in red.

Raddose-3D script for the X-ray dose calculation for 1x1x1 µm crystals

```
######################################################################
##########
#                               Crystal Block
#
######################################################################
##########
Crystal
Type Cuboid
PixelsPerMicron 2
Dimensions 1 1 1
AbsCoefCalc RD3D
UNITCELL 79.2 79.2 37.8 90 90 90
ANGLEP 0
```

```
ANGLEL 0
NumMonomers 8
NumResidues 129
ProteinHeavyAtoms S 4
SolventFraction 0.59
######################################################################
##########
#                               Beam Block
#
######################################################################
##########
Beam
Type Gaussian
FWHM 9 4
Collimation Rectangular 27 12
FLUX 8.6e12
ENERGY 12.0
######################################################################
##########
#                               Wedge Block
#
######################################################################
##########
Wedge 0 1
ExposureTime 7.69E-3
ANGULARRESOLUTION 0.01
STARTOFFSET -1.8 0 0
TRANSLATEPERDEGREE 3.6 0 0
ROTAXBEAMOFFSET 0
```

Raddose-3D script for the X-ray dose calculation for 5x5x5 µm crystals

```
######################################################################
##########
#                               Crystal Block
#
######################################################################
##########
Crystal
Type Cuboid
PixelsPerMicron 2
Dimensions 5 5 5
AbsCoefCalc RD3D
UNITCELL 79.2 79.2 37.8 90 90 90
ANGLEP 0
ANGLEL 0
NumMonomers 8
NumResidues 129
ProteinHeavyAtoms S 4
SolventFraction 0.59
######################################################################
##########
#                               Beam Block
#
```

```
######################################################################
##########
Beam
Type Gaussian
FWHM 9 4
Collimation Rectangular 27 12
FLUX 8.6e12
ENERGY 12.0
######################################################################
##########
#                                Wedge Block
#
######################################################################
##########
Wedge 0 1
ExposureTime 7.69E-3
ANGULARRESOLUTION 0.01
STARTOFFSET -1.8 0 0
TRANSLATEPERDEGREE 3.6 0 0
ROTAXBEAMOFFSET 0
```
